# Psychotropic drug use as indicator of mental health in adolescents affected by a plexus injury at birth: A large population-based study in Sweden

**DOI:** 10.1371/journal.pone.0193635

**Published:** 2018-03-21

**Authors:** Elia Psouni, Raquel Perez Vicente, Lars B. Dahlin, Juan Merlo

**Affiliations:** 1 Department of Psychology, Faculty of Social Sciences, Lund University, Lund, Sweden; 2 Unit for Social Epidemiology, Department of Clinical Sciences (Malmö), Faculty of Medicine, Lund University, Malmö, Sweden; 3 Department of Translational Medicine - Hand Surgery, Faculty of Medicine, Lund University, Lund, Sweden; 4 Department of Hand Surgery, Skåne University Hospital, Malmö, Sweden; Vanderbilt University, UNITED STATES

## Abstract

Chronic handicap in early life may have a long-term impact on children’s psychosocial well-being. Here, we investigated whether Brachialis Plexus Birth Injury (BPBI)—an unpredictable injury at birth—is associated with worse mental health later on, as indicated by prescription and use of psychotropic drugs in adolescence. We explored further whether this association is different depending on socioeconomic characteristics of the child’s family, as well as sex. Of the 641 151 children born to native parents in Sweden 1987–1993 (alive and still living in Sweden at the end of 2008), identified in the *Swedish Medical Birth Registry*, 1587 had suffered a BPBI. Logistic regression analysis was performed to assess the impact of socioeconomic characteristics and associations with later psychosocial health. Results show that beyond the known increased risks for females as compared to males, BPBI, but also lower family income, further increased the risk of burdened mental health requiring psychotropic drug use in adolescence. The effects were additive. Thus, compared to unaffected peers, teenagers who suffered a BPBI at birth are at higher risk of suffering poor mental health during adolescence, independently of surgical intervention and its outcome. Girls growing up in families with lower socioeconomic status have this risk added to their already increased risk of poor mental health during adolescence.

## Introduction

Child chronic illness may cause deep, long-lasting crisis in affected families [1–3]. Long-term effects on psychosocial wellbeing and further development are frequent, with behavioral and emotional manifestations on affected children’s and their parents’ mental health [4–5]. Indeed, parents coping with child chronic illness or injury/handicap are under considerable stress, particularly if problems start already at the child’s birth [6–9].

One handicapping condition with onset at birth is *Brachial Plexus Birth Injury* (BPBI), most often caused by strain to the brachial plexus nerve roots between head and shoulder during the downward traction of the fetus’ head during birth. The injury may affect the upper part only (C5-C6, sometimes C7 spinal nerve roots) or involve the entire brachial plexus (C5-Th1), with subsequent variable degree of arm and hand dysfunction [10]. Evidence regarding prevalence of BPBI varies considerably. For example, population-based studies using the Swedish Medical Birth Registry 1980–1997 over periods from 9 to14 years, have reported steadily increasing incidences of BPBI, from 1.4 cases [11] to 2.3 cases [10] to 2.7 cases per 1000 live births in 1997 [12], to 3.2 cases per 1000 live births [13]. By contrast, a large epidemiological study in USA reported lower occurrence of BPBI, about 1.5 cases per 1000 births over the period 1997–2003 [14]. Heavy birth weight is discussed as the strongest risk factor for a BPBI injury [12, 15] although over 50% of BPBI cases in early analyses were neither related to birth trauma nor to heavy birth weight [16]. More recent analysis, based on about 11 million births in the USA, corroborates these early findings: while exceptionally large birth weight (>4.5 kg) gave 14 times greater risk, and forceps delivery 9 times greater risk, for BPBI, known obstetric risk factors were present in only 46% of all BPBI cases [14].

The prognosis for BPBI-injured children varies depending on location and extent of the injury. Injuries affecting the entire brachial plexus (C5-Th1) usually require nerve reconstruction and less than 40% of children regain function of the arm and hand entirely. Prognosis for C5-C6 (C7) BPBI is significantly better, with about 80–90% of children regaining function [13]. However, as it is not possible to predict, in the single case [17], the consequences of an injury [13, 16], parents are commonly informed that their child may develop neurological disability of unpredictable degree. Combined with the fact that the child seemed entirely healthy until birth, but unexpectedly suffered a disabling injury at birth, this unpredictability and the necessary wait for a more accurate prognosis [17], may be additional stressors for parents [18–19]. It is therefore not surprising that BPBI diagnosis is often linked with parental dissatisfaction concerning both the birth and consequent contacts with the healthcare system [10, 20]. In such contacts, dissatisfaction appears to stem from the communication with medical staff, particularly the parents’ perceived unmet needs for information concerning the injury [19–20] and how to optimally care for the injured baby [10]. Importantly, early parental experiences of children with a BPBI are negative regardless of the severity of the child’s injury [20–21], as is the occurrence of later maternal depression and anxiety [22], suggesting that the degree of parental distress may depend on other factors than the injury itself. Importantly, the possibility that these experiences are modified by socioeconomic circumstances of the family has not been explored.

Although physical abnormalities affecting the face or hands are known to have a strong impact on individual psychological well-being [21, 23], the behavioral and developmental consequences of BPBI have been largely overlooked [24]. The little evidence regarding psychological health in children that have suffered a BPBI is inconsistent. Based on 44 children with BPBI of varying severity, an early study [20, 25] indicates that over 50% scored above cut-off for behavior problems requiring further attention, a frequency higher than published norms for non-psychiatric populations. Children whose injuries had required surgery had a significantly poorer developmental outcome than children with milder injuries, but the most significant difference between these groups concerned self-esteem and social development [20]. However, a more recent study based on 31 children found no conclusive evidence of negative self-concept or poor socioemotional functioning, although girls seemed more vulnerable than boys [24]. These findings are inconclusive regarding potential behavioral and developmental consequences of BPBI beyond the motor sphere, but together suggest that different groups of families could be affected differently. In addition, the studies were based on small samples [20, 24–25], selected patient populations and mostly self-reported information obtained by affected children, parents or teachers, so generalizability is limited. Furthermore, data when affected children have reached adolescence, an emotionally turbulent period when peer acceptance and independence achievement become increasingly important [26], are entirely missing.

### The present study

To our knowledge, no large population study has adopted a life-course approach [27] to investigate the potential impact of BPBI on psychological health in adolescence. Therefore, the central aim of the present investigation was to describe the psychological health of adolescents with BPBI. Contributions of BPBI on adolescent use of psychotropic medication were assessed, considering use of psychotropic medication as a surrogate of impaired psychological health. This approximation has been used in other research [28–30] and seems appropriate within homogenous and accessible healthcare systems, such as the Swedish. As socioeconomic characteristics may impair the potential capacity to cope with BPBI-related distress [24], and it also known that girls suffer higher levels of psychological distress and consume more psychotropic drugs than boys [31], we adopted an intersectional approach [32] and explored possible interactions of BPBI with those variables in relation to use of psychotropic medication. Furthermore, we not only investigated whether BPBI and its interaction with socioeconomic factors and gender increased the probability of use of psychotropic medication in adolescence, but also whether this information could be an appropriate index for distinguishing future users of psychotropic medication from non-users [33–34]. For the purpose of our investigation, we used several Swedish nationwide healthcare registers that continuously record individual level information.

## Methods

### Study population

**Fig 1:**
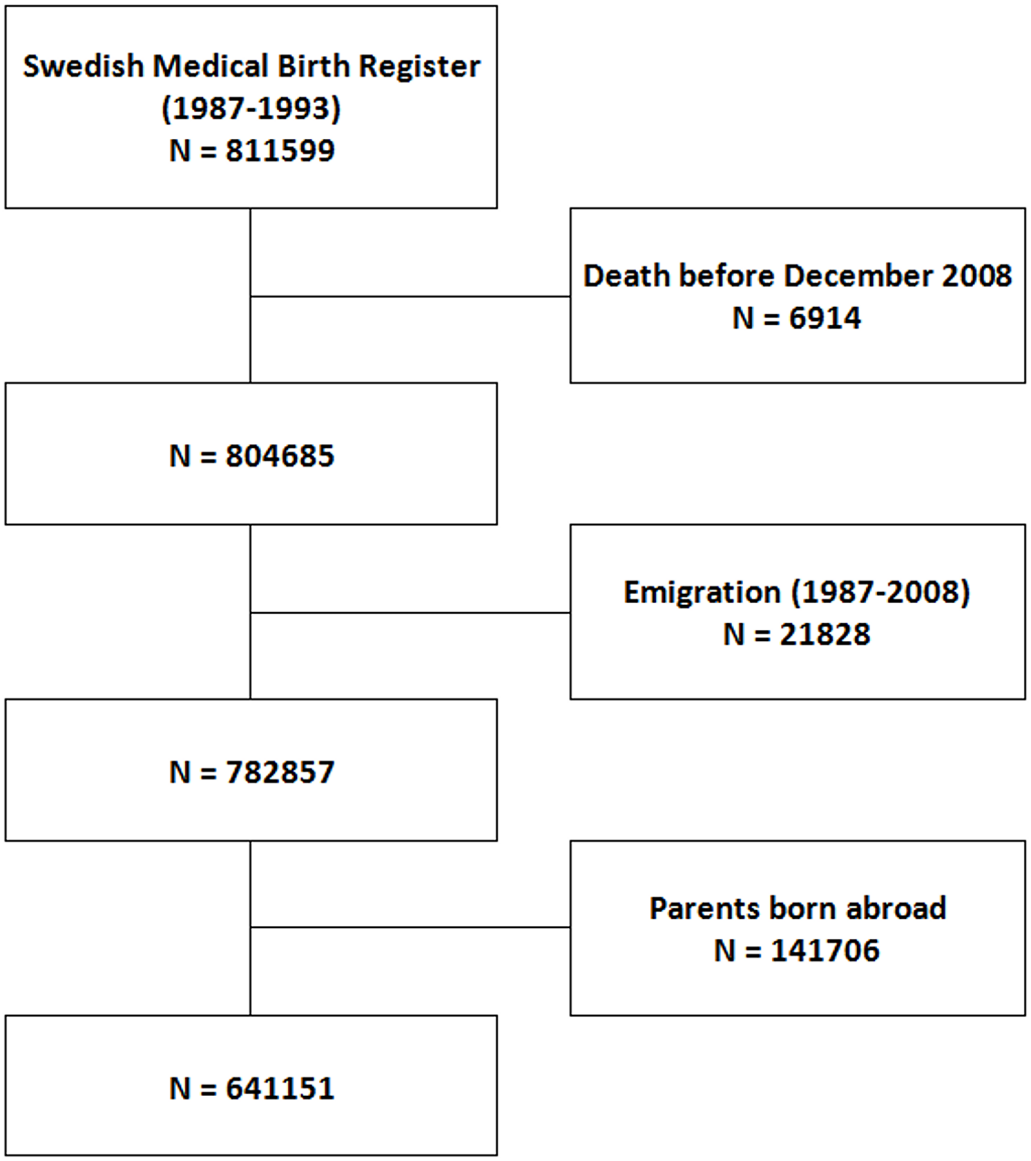
Study population.

A database was obtained, derived from the *Swedish Medical Birth Register* linked to other national databases, such as the *Swedish Drug Prescription Register*, the *National Mortality Register*, the *Emigration Register* and the *National Inpatient Register*, all administered by Statistics Sweden and by the National Board of Health and Welfare. In the obtained data, identification numbers were replaced with arbitrary numbers, enforcing anonymity. All children born in Sweden during the period 1987–1993 were identified (811 599 children). After excluding children who had died or emigrated before 31 December 2008, the cohort was reduced to 782 857 children. Given evidence of relative underuse of psychotropic drugs in relation to needs in adolescent descendants of migrant women [29], children of parents born outside Sweden were excluded in order to avoid potential information bias. The final cohort consisted of 641 151 children/adolescents between 12 and 21 years of age (Fig 1). The database was approved by the Regional Ethical Review Board in South Sweden.

### Assessment of variables

The main purpose was to investigate the association between BPBI and use of psychotropic drugs in adolescence. Adopting an intersectional approach [32], we also examined familial socioeconomic position and the adolescent’s gender as possible effect modifiers of this association. For instance, the association between BPBI and psychotropic drug use in girls from families with low socioeconomic position might be stronger than the simple additive effects of BPBI, gender and low socioeconomic position. We also aimed at identifying variables that could be a common cause of both BPBI and psychotropic drug use and, thereby, confound the association between BPBI and psychotropic drug use. For this purpose, we first considered variables which, based on previous findings, are associated with increased risk for BPBI [12–15, 17]. Thereafter, we considered these variables’ potential association with psychotropic drug use in adolescence, thereby distinguishing between variables used in interaction analyses and potential confounders.

#### Outcome variable: Psychotropic drug use

The Swedish Drug Prescription Register contains all drugs dispensed at Swedish pharmacies in outpatient settings since July 2005. Information concerning medication use is systematically obtained from all the Swedish pharmacies in a computerized, standardized way. All dispensations are recorded–so the information is not based on prescriptions, but on actually obtaining the medication at the Pharmacy. Prescribed and received medication within hospitals and nurse homes for the elderly is not included in the database [35]. Five psychotropic drug categories, according to the Anatomical Therapeutic Chemical (ATC: [36]) classification system, were considered: antipsychotic (N05A), anxiolytic (N05B), hypnotic and sedative (N05C), antidepressant (N06A) and psycho-stimulant (N06B). The register contains individual information on medication starting1^st^ July 2005, which conditions the period of analysis for this study. Psychotropic drug use was thus operationalized as “at least one dispensed prescription of drugs from any of the categories above, for the period 1^st^ July 2005 to 31^st^ December 2008” (yes/no).

#### Main exposure variable: BPBI

We identified all children recorded in the Patient Registry with a hospital discharge diagnosis of *BPBI* according to the following ICD10 (P14.0, P14.1, P14.2, P 14.3, P14.8, P14.9 and T92.4) or ICD9 codes (767.4, 767.6, 767.7, and 907.4) [37]. Although most cases are identified early after birth, we conducted a follow up one year after delivery (until 2004) in order to identify diagnoses of late effect of injury to peripheral nerve of shoulder girdle and upper limb (codes 907.4 and T92.4). We also identified cases of *birth trauma* in general (ICD10 code P14 and ICD-9 code 767), such as trauma-like injuries to scalp, fracture of clavicle, facial nerve injury, eye damage and distinguished those from BPBI. This variable was used in a complementary analysis to investigate possible associations between birth trauma in general and use of psychotropic drugs in adolescence and, distinguishing between BPBI and other types of trauma.

#### Variables in interaction analyses

**Sex**: Sex differences have been reported in occurrence of BPBI, with boys being more at risk [13], and with respect to use of psychotropic drugs, with girls in general consuming more than boys [28, 31]. It has also been suggested that girls with BPBI may be at a higher risk of negative consequences than boys with BPBI [22].

**Familial socioeconomic position**: Low socioeconomic position is associated with increased use of psychotropic drugs in adolescents [38] but it is unknown whether it is related to risk for BPBI. We operationalized socioeconomic position using information on equalized household income from both mother and father, the year before the child’s birth [39–41]. In a first step, each parent’s income was categorized in deciles. Then, the mother’s income was reclassified as the highest decile value in the couple (e.g. if the mother was in decile group 4 and the father in decile group 10, the mother was reclassified to the decile group 10). Low socioeconomic position was defined as having a household income in decile group 4, or lower.

**Interaction variable**: We created a categorical variable combining BPBI, sex and income and used the category of boys without BPBI from families with high socioeconomic position as reference.

#### Potential confounders

**Birth weight**: Birth weight was included based on evidence that it is implicated in risk for both BPBI [14] and impaired mental health during adolescence [41]. We categorized birth weight as (a) < 3000 gr, (b) 3000–3499 gr, (c) 3500–3999 gr, (d) 4000–4499 gr and (e) > 4500 gr at birth.

**Gestational age** was included as it may possibly increase the risk for both BPBI [13, 17] and impaired mental health [42–43]. Therefore, we defined gestational categories (a) <28 weeks, (b) 28–37 weeks, (c) 37–40 weeks, (d) 40–42 weeks and (e) >42 weeks.

**Mode of delivery** was considered based on evidence of associations to both BPBI [14] and adolescent mental health [43]. Four categories indicating mode of delivery were included: (a) spontaneous vaginal, (b) cesarean section, (c) operative vaginal, (d) vacuum or forceps and (e) combined, where several modes occurred.

**Apgar score at 5 minutes**: The Apgar score is considered an objective way of describing the infant’s adaptation to extra-uterine life [44], based on assessment of heart rate, respiratory effort, muscle tone, reflex irritability, and color. A low Apgar score may be regarded as a possible proxy of an underlying condition related to BPBI [45] and may confound the association between BPBI and use of psychotropic drugs in adolescence. We used the Apgar score at 5 minutes (Apgar5), with following amendments: When Apgar 1 was available and high, and there was no Apgar information at 5 or 10 minutes, we set the Apgar 5 value at > = 7 (high Apgar1 values often result in that no further Apgar assessments being recorded). If Apgar 10 was available, but Apgar 5 was missing, we set it at <7. Apgar information was entirely missing for 4862 children. Missing Apgar score was not associated with BPBI or with worse adolescent mental health.

**Maternal smoking**: The mother’s self-reported smoking habits when first assigned to antenatal care (between 8^th^ and 12^th^ gestational week) was considered. We categorized smoking habits into (a) no smoking, (b) light smoking (1–9 cigarettes per day), (c) heavy smoking (>9 cigarettes per day) and (d) missing information (N = 37 477). The non-smoking group was set as reference. Maternal smoking reduces birth weight and may be related to both prolonged labor wit risk for BPBI [46] and to the use of psychotropic drugs in adolescence [47].

**Diabetes Mellitus**: There is previous evidence of association between maternal diabetes and increased risk for BPBI [48]. We hypothesized that maternal diabetes might also condition used psychotropic drugs in adolescence and considered, therefore, a dichotomous variable: (a) no maternal diabetes (b) maternal diabetes.

**Multiple births**: Individuals born in multiple births might have a different risk of developing impaired mental health later in life, compared to singletons, but also of suffering a BPBI [14, 45]. In fact, many previous studies have restricted their analysis to singletons [49–50]. We therefore considered this variable as a confounder.

### Statistical analysis

We applied logistic regression analysis to obtain odds ratios (OR) and 95% confidence intervals. Using simple models we first quantified the association between each of the variables considered as potential confounder of the risk of suffering BPBI and of using psychotropic drugs in adolescence (Table 1). Next, in the intersectional analysis we investigated the interaction between BPBI, sex and familial income on use of psychotropic drugs in adolescence (Table 2). To quantify the existence of interaction, we calculated the excess risk due to interaction (RERI) and the synergy index (SI) with 95% confidence intervals, using the Epinet’s Excel sheet [51] that is based on a method by [52]. The reference category was boys from families with high income and without BPBI, and the interaction category was girls with low income suffering from BPBI.

**Table 1:** Characteristics of the population in relation to presence of brachial plexus injury at birth and psychotropic drug use in adolescence. Values are number of cases/number of individuals (percentage) as well as odds ratios (OR) and 95% confidential interval (CI).

	Brachial Plexus Birth Injury 1587/641151 (0.25%)		Use of psychotropic drugs in adolescence 46271 /641151 (7.22%)	
		OR (95% CI)		OR (95% CI)
*Sex*				
Girls	715/311917 (0.23)	0.87 (0.78–0.96)	26889/311917 (8.62)	1.51 (1.48–1.54)
Boys	872/329234 (0.26)	Ref	19382/329234 (5.89)	Ref
*Income*				
High	1233/485801 (0.25)	Ref	31907/485801 (6.57)	Ref
Low	354/155171 (0.23)	0.90 (0.80–1.01)	14351/155171 (9.25)	1.45 (1.42–1.48)
Missing information	0/179 (0.0)	Nc	13179 (7.26)	1.11 (0.63–1.95)
*Singleton*				
Yes	1568/626107 (0.25%)	Ref	45325/626107 (7.24%)	Ref
No	19/15042 (0.13%)	0.50 (0.32–0.79)	945/15042 (6.28%)	0.86 (0.80–0.92)
*Smoking*, *first maternal-care visit*				
No	1180/454418 (0.26%)	Ref	28786/454418 (6.33%)	Ref
Yes	284/148035 (0.19%)	0.74 (0.65–0.84)	14258/148035 (9.63%)	1.58 (1.54–1.61)
Missing information	123/38698 (0.32%)		3227/38698 (8.34%)	1.35 (1.30–1.40)
*Maternal Diabetes Mellitus*				
No	1556/638587 (0.24%)	Ref	46036/638587 (7.21%)	Ref
Yes	31/2564 (1.21%)	5.01 (3.50–7.16)	235/2564 (9.17%)	1.30 (1.14–1.49)
*Mode of delivery*				
Spontaneous vaginal delivery	1083/518934 (0.21%)	Ref	37066/518934 (7.14%)	Ref
Cesarean section	76/68206 (0.11%)	0.53 (0.42–0.67)	5513/68206 (8.08%)	1.14 (0.11–1.18)
Vacuum or forceps	377/33691 (1.12%)	5.41 (4.81–6.09)	2510/33691 (7.45%)	1.05 (1.00–1.09)
Operative vaginal delivery	12/11972 (0.61%)	3.69 (1.65–5.18)	110/1972 (5.58%)	0.77 (0.63–0.93)
Combined	39/18348 (0.21%)	1.02 (0.74–1.40)	1072/18348 (5.84%)	0.81 (0.76–0.86)
*Apgar Score at 5 minutes*				
<7	108/5593 (1.93%)	8.39 (6.89–10.21)	599/5493 (10.71%)	1.55 (1.43–1.69)
≥7	1465/625750 (0.23%)	Ref	44873/625750 (7.17%)	Ref
Missing information	14/9808 (0.14)	0.61 (0.36–1.03)	799/9808 (8.15)	1.15 (0.77–1.23)
*Birth weight (g)*				
≤ 3000	91/93236 (0.10%)	1.08 (0.84–1.39)	7946/93236 (8.52%)	1.15 (1.12–1.18)
3000–3499	181/200714 (0.09%)	Ref	15072/200714 (7.51%)	Ref
3500–37999	420/225433 (0.19%)	2.07 (1.74–2.46)	15261/225433 (6.77%)	0.89 (0.87–0.92)
4000–4499	485/98783 (0.49%)	5.46 (4.61–6.49)	6463/98783 (6.54%)	0.86 (0.94–0.89)
>4500	407/22440 (1.81%)	20.47 (17.17–24.40)	1478/22440 (6.59%)	0.87 (0.82–0.92)
Missing information	3/545 (0.55%)	6.13 (1.95–19.25)	51/545 (9.39%)	1.27 (0.95–1.70)
*Gestational age (weeks)*				
<37	49/37146 (0.13%)	0.70 (0.52–0.93)	3201/37146 (8.62%)	1.20(1.16–1.25)
37 and <40	506/266809 (0.19%)	Ref	19375/266809 (7.26%)	Ref
≥40 and <42	840/291755 (0.29%)	1.52 (1.36–1.70)	20397/291755 (6.99%)	0.96 (0.94–0.98)
≥42	190/44545 (0.43%)	2.25 (1.91–2.66)	3205/44545 (7.19%)	0.99 (0.95–1.03)
Missing information	2/896 (0.22%)	1.18 (0.29–4.73)	93/896 (10.38%)	1.48 (1.19–1.83)
*Birth trauma*				
No	0/624391 (0.00)	Nc	45062/624391 (7.22)	Ref
Only BPBI	1558/1558 (100.00)	Nc	139/1558 (8.92)	1.26 (1.06–1.50)
Other trauma	0/15173 (0.00)	Nc	1069/15173 (7.05)	0.97 (0.92–1.04)
Both	29/29 (100.00)	Nc	1/29 (3.45)	0.46 (0.06–3.37)

Notes: Ref = Reference group. Nc = Non calculable.

**Table 2:** Psychotropic drug use in adolescence in relation to presence of BPBI, offspring sex and maternal income level. Values are odds ratios (OR) and 95% confidence intervals (CI).

	*Model 1*	*Model 2*	*Model 3*	*Model 4*	*Model 4 adjusted* *
Girls vs. boys	1.51 (1.48–1.54)				
BPBI: Yes vs. no		1.24 (1.05–1.48)			
Income: High vs. low			1.45 (1.42–1.48)		
No BPBI-high income-boy				Ref	Ref
No BPBI-Low income-boy				1.39 (1.35–1.43)	1.38 (1.33–1.42)
BPBI-high income-boy				1.34 (1.00–1.80)	1.33 (1,00–1,79)
BPBI -Low income-boy				2.06 (1.31–3.24)	2.04 (1.29–3.20)
No BPBI-high income-girl				1.48 (1.44–1.51)	1.47 (1.43–1.50)
No BPBI- Low income-girl				2.21 (2.15–2.27)	2.17 (2.11–2.24)
BPBI-high income-girl				1.87 (1.41–2.47)	1.89 (1.43–2.51)
BPBI-Low income-girl				2.15 (1.30–3.57)	2.20 (1.33–3.65)
Au-ROC (95% CI)	0.551 (0.549–0.553)	0.500 (0.500–0.501)	0.537 (0.535–0.539)	0.569 (0.567–0.571)	0.591 (0.589–0.594)

*Model adjusted for: singleton, maternal smoking, diabetes, mode of delivery, Apgar, gestational age, weight.

In the latter models we also calculated the area under the receiver operating characteristic (AU-ROC) curve as indication of discriminatory accuracy. The ROC curve is constructed by plotting sensitivity or the true positive fraction (TPF) against 1-specificity or false positive fraction (FPF) [53–54] and quantifies discriminatory accuracy [53–56]. An AU-ROC value of 0.5 indicates complete lack of discriminatory accuracy, and thus a useless model, while a value of 1.0 indicates complete discriminatory accuracy. IBM SPSS Statistics for Windows, Version 20.0 and STATA version 14.1 were used for statistical analysis.

## Results

Overall, 1587 children with BPBI were identified, ca 2 per 1000 births, while ca 72 per 1000 used psychotropic drugs in adolescence (Table 1). Children with BPBI were compared to other children in the Birth Register. Risk factors associated with the child’s birth are summarized in Table 1, separately for males and females. Higher occurrence was associated with diabetes mellitus in the mother, a higher gestational age, the use of vacuum or forceps at delivery and operative delivery. The birth characteristics associated with highest risk for BPBS were a birth weight above 4500 gr and a characterization of the newborn as large for gestational age.

### Population characteristics related to BPBI and use of psychotropic drugs

As expected, possible confounders were associated with higher risk for both BPBI and use of psychotropic drugs in adolescence. For instance, compared to singleton births, multiple birth deliveries decreased the risk of BPBI (possibly as a result of cesarean delivery and lower birth weights in multiple births) and also the risk of using psychotropic drugs in adolescence. However, while cesarean delivery was associated with decreased risk of BPBI, it was associated with slightly higher risk of use of psychotropic drugs in adolescence. Vacuum or forceps increased five times the risk of BPBI, but had a weak influence on the risk for psychotropic drug use. As expected, the risk of BPBI increased exponentially with offspring birth weight. However, a higher weight at birth was associated with less risk for use of psychotropic drugs in adolescence, compared to a lower weight at birth. Maternal diabetes increased considerably the risk of BPBI (by a factor of 5), but children of diabetic mothers had only slighter higher risk for using psychotropic drugs in adolescence. An Apgar score <7 represented much higher risk of BPBI and also higher risk of psychotropic drug use in adolescence. Concerning the variables considered as effect modifiers, neither child sex nor household income was associated to BPBI. However, low income and being a girl increased the risk of using psychotropic drugs.

### Association between BPBI and use of psychotropic drugs and interaction analysis

Children with BPBI had a higher risk of using psychotropic drugs as adolescents (OR = 1.24, 95%CI: 1.05–1.48) compared to children without a BPBI (Table 2). Also, while BPBI was associated to increased risk of use of psychotropic drugs in adolescence, other forms of birth injury were not (Table 2). Setting as reference for comparisons boys without BPBI and from families with high income, low income girls with BPBI were in double as high risk of using psychotropic drugs (OR = 2.15, 95%CI:1.30–3.57). This increased risk was, however, almost identical to the risk of using psychotropic drugs among low-income girls without BPBI (OR = 2.21, 95%CI: 2.15–2.27), suggesting that there is no additional risk increment beyond what each of these factors contributes. Findings were similar for boys. Thus, results indicate absence of interaction between BPBI, child sex and family income concerning risk for psychotropic drug use in adolescence. This observation was confirmed in the RERI and SI calculations.

**Fig 2:**
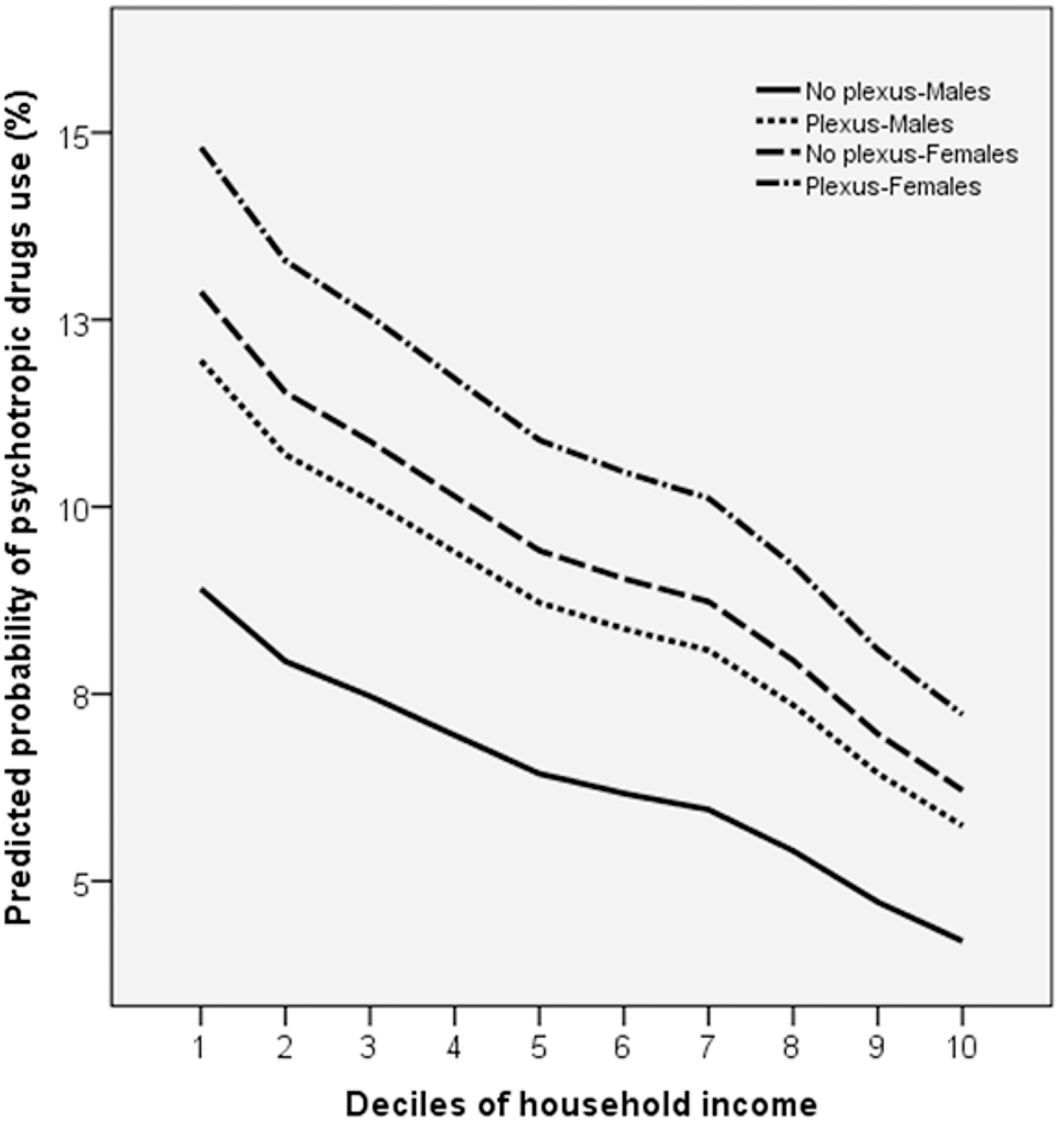
Predicted probability of psychotropic drug use in adolescents by individualized household income, gender and Brachial Plexus Birth Injury (BPBI).

**Fig 3:**
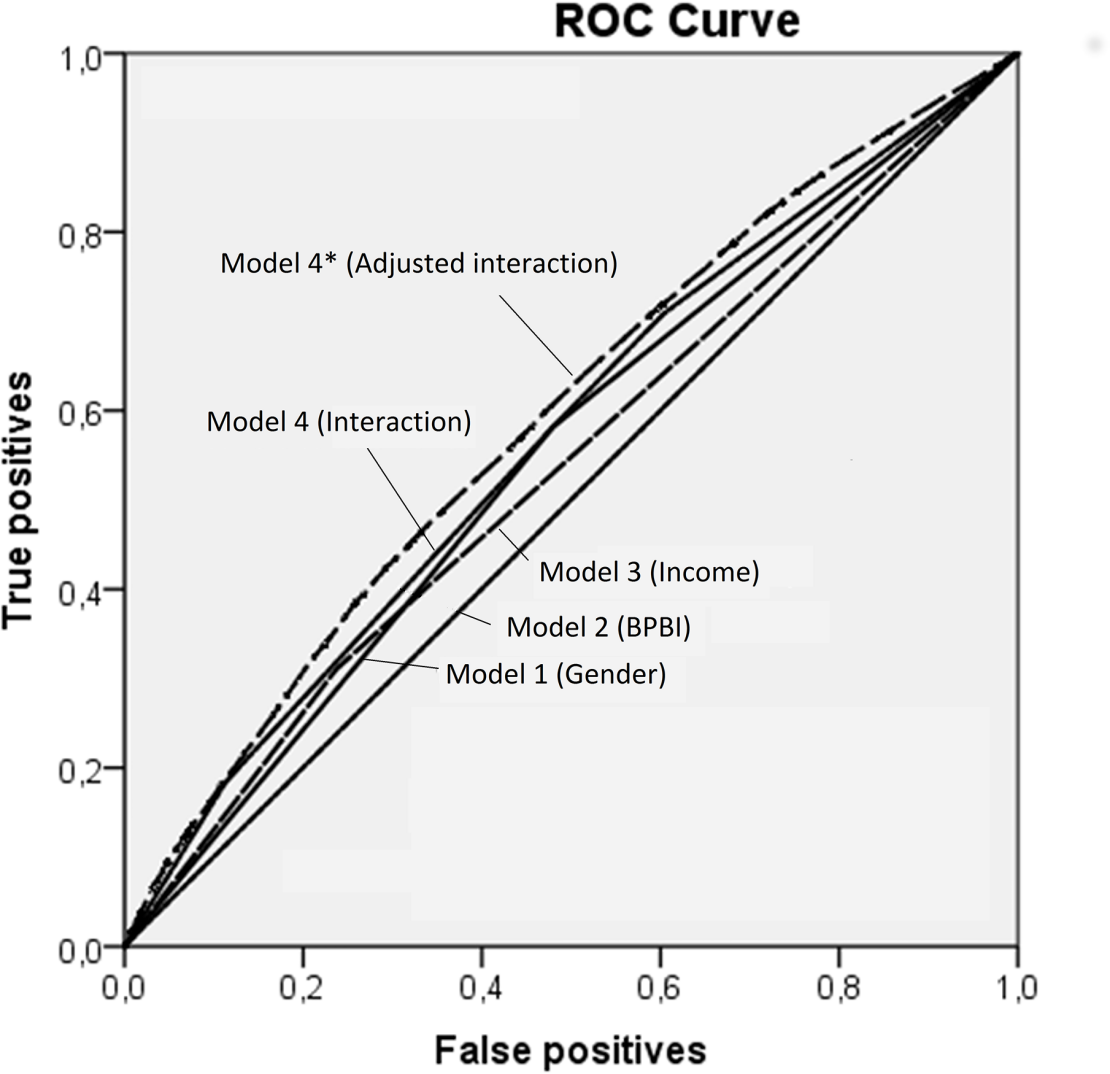
ROC-areas: Sensitivity and specificity of the models.

Fig 2 illustrates these associations using a finer categorization of household income, in decile groups. The predicted probability of using psychotropic drugs continuously decreases as household income increases. However, at every decile group of income, the risk of psychotropic drug use is higher for girls compared to boys and higher for adolescents who suffered a BPBI compared to those who did not. The interaction between BPBI, sex and household income did not increase the accuracy of the model for discriminating between adolescents who had and those who had not been prescribed and used psychotropic drugs, as the increase in the AU-ROC was only 0.032 units and 0.091 units higher than chance (Table 2, Fig 3). In addition, BPBI alone did not have any discriminatory accuracy.

## Discussion

Our analyses, based on a large population database covering the whole of Sweden, indicate that, on average, children who suffered a BPBI are at a higher risk of using psychotropic medication in adolescence, compared to the group of unaffected children. Since prescription–and therefore use–of psychotropic medication is a clear indicator of psychological health impairment, these findings suggest that adolescents with a BPBI may be in higher risk for impaired mental health. Therefore, our analyses confirm previous findings that children with BPBI injury have more difficulties in psychosocial adjustment compared to their peers without such an injury [20, 25], extending this finding to adolescence. Previous studies reported that, as developmental and behavioral complications associated with the BPBI had not been identified and reported earlier, most children were not receiving appropriate help (e.g., [57]). On the other hand, it cannot be excluded that the closer follow-up of children with BPBI and associated handicap by medical providers may result in a higher rate of detection and medication treatment for psychiatric concerns, compared to detection rates in the general population.

Importantly, the BPBI group in our analysis was not differentiated with respect to severity of injury or nature of consequent handicap and reflects, therefore, the entire range of BPBI-consequences. Thus, our findings have important clinical implications. As is the case for other hand injuries, continuous and long-term follow-up of the children with BPBI is crucial [58] in order to properly assess the degree to which the child has function of his/her arm, particularly frequently observed shoulder problems [59]. It appears that continuous and long-term follow up is also necessary in order to secure that the information that parents–and, later on, also children–are receiving is sufficient and satisfactory [60], that parental concerns how to best support the child’s development are responded to [22], and that the child’s overall motor, cognitive but also socioemotional functioning is followed up as recommended for children with a traumatic peripheral nerve injury [61]. Our findings also suggest that following the child’s own concerns about their handicap, irrespective of severity, may also be important [4]. As considerable emotional pressure is put on parents even when professional information about the condition is provided, they and their children who suffered a BPBI may need to receive support from health care professionals focusing on psychosocial coping, addressing consequent concerns about the children’s handicap and wellbeing with equal gravity as when a child is handicapped by other chronic conditions [1, 9, 22, 60–62].

Our data highlight that, compared to male adolescents, female adolescents were at higher risk of being prescribed and using psychotropic drugs, consistent with previous evidence [28, 31], and it is also evident that this pattern is stable across different levels of family income. Given this, BPBI was associated with higher risk of psychotropic drug use suggesting compromised mental health across all income groups within each gender, while other forms of birth injury were not. The increment in risk associated with BPBI was highest for boys with low family income (43%), compared to high family income boys and girls (30%). Notably, the increment in risk of being prescribed and using psychotropic drugs during adolescence associated with being a girl was larger than that of having suffered BPBI and BPBI added no risk for the already most vulnerable group of girls from low income families.

Observing a “significant” association between an exposure variable of interest (here a BPBI) and an individual health outcome (here the prescription and thereby use of psychotropic drugs) alongside with a tiny discriminatory accuracy (indicated by the AU-ROC close to .50 found in our study) may be confusing. However, while a statistically significant association is always relevant from a probabilistic approach, it may not be helpful from a mechanistic point of view [33]. This apparent paradox can be better understood considering that the use of measures of discriminatory accuracy in epidemiological studies is completely analogous to the use of such measures in other fields, such as the study of biomarkers and diagnostic tests [34, 54, 63]. It is well recognized that, although significantly associated with higher risk for a disease, many novel biomarkers are essentially not useful because they have a very low discriminatory accuracy [54]. In order to increase the discriminatory accuracy and, thereby, the causal strength of observational “risk factors”, we would need to identify individuals, or homogeneous groups of individuals, with clearly increased susceptibility to a particular outcome from a given exposure. Adopting this approach, however, involves decisions concerning meaningful groupings. As a first step in the present study, we explored a possible interaction between BPBI, sex and household income. Given that this approach did not produce a model with satisfactory discriminatory power, it may be necessary in future studies to use clinical or large-scale databases with detailed information concerning also individual psychosocial functioning. Improving the discriminatory accuracy in epidemiological studies is a fundamental challenge in future epidemiology [33, 62, 64].

Our findings indicate that obstetric variables, including vacuum and forceps delivery, but also operative vaginal delivery, infant birth weight and gestational age over 42 weeks, are associated with increased risk for a BPBI, confirming previous epidemiological research [12–15]. In fact, in the present cohort, a birth weight between 4000 and 4499 gr increased the risk for a BPBI with over 8 times, while a birth weight above 4500 was associated with a 36 times higher risk of a BPBI. Thus, high birth rate was present in 65.6% of BPBI in the database. Previous epidemiologic analyses, based on the same cohort, appear to have overlooked the large amounts of missing data on a number of critical variables, reporting effect values that may be misleading.

Our study has limitations. To begin with, while use of psychotropic medication is a clear indicator of poor psychological health, other possible treatments of poor mental health commonly used with children and adolescents, such as psychotherapeutic intervention, were not considered here as no information on such treatments was available in the databases. This may have resulted in an underestimation of poor mental health in all populations considered here. If, in addition, more children handicapped by a BPBI have ongoing contacts with professionals to whom they can turn when experiencing psychosocial problems, there is a risk that our analyses suffer differential information bias, particularly among the more severely handicapped youth. Finally, although the percentage of children with BPBI is small at a population level, the fact that the population of children who did not suffer a BPBI was not restricted to children without other known birth defects may have resulted in residual confounding. Also, as all information used in this study was collected from registries using only the ICD-9 and the ICD-10 codes, and thus not confirmed by a clinician in order to check the correctness of diagnosis, it cannot be excluded that some cases were misclassified.

### Conclusion

Suffering a brachial plexus injury at birth can increase the risk of impaired psychological health in adolescence. This increased risk is present along all different family economic levels and equally for boys and girls. Hence, children with a BPBI, and their parents, are in need of more attention and effective psychological support than in current praxis, in order to assist a prevention of long term adverse consequences of the initial injury. Our findings have a clear theoretical impact for further research; it is important to identify the qualities that promote good adjustment and psychosocial wellbeing of affected children and their parents [22, 24, 61, 65–66], as children’s ability to manage emotions and adjust in times of difficulty is affected by the developmental environments they grow into. A better understanding of the developmental environments of children affected by chronic handicap in early life is thus essential for preventing the spiral of decline in mental health for the child, moderating the suffering of affected children and their families.
